# The Omic Insights on Unfolding Saga of COVID-19

**DOI:** 10.3389/fimmu.2021.724914

**Published:** 2021-10-20

**Authors:** Arvinpreet Kaur, Mehak Chopra, Mahak Bhushan, Sonal Gupta, Hima Kumari P, Narmadhaa Sivagurunathan, Nidhi Shukla, Shalini Rajagopal, Purva Bhalothia, Purnima Sharma, Jalaja Naravula, Renuka Suravajhala, Ayam Gupta, Bilal Ahmed Abbasi, Prittam Goswami, Harpreet Singh, Rahul Narang, Rathnagiri Polavarapu, Krishna Mohan Medicherla, Jayaraman Valadi, Anil Kumar S, Gyaneshwer Chaubey, Keshav K. Singh, Obul Reddy Bandapalli, Polavarapu Bilhan Kavi Kishor, Prashanth Suravajhala

**Affiliations:** ^1^ Department of Bioinformatics, Hans Raj Mahila Maha Vidyalaya, Punjab, India; ^2^ Bioclues.org, Hyderabad, India; ^3^ Centre for Bioinformatics, School of Life Sciences, Pondicherry University, Puducherry, India; ^4^ Department of Biological Sciences, Indian Institute of Science Education and Research, Kolkata, India; ^5^ Department of Biotechnology and Bioinformatics, Birla Institute of Scientific Research, Jaipur, India; ^6^ Vignan’s Foundation for Science, Technology & Research (Deemed to be University), Guntur, India; ^7^ Department of Chemistry, School of Basic Sciences, Manipal University Jaipur, Jaipur, India; ^8^ Functional Genomics Unit, Council of Scientific and Industrial Research- Institute of Genomics & Integrative Biology (CSIR-IGIB), Delhi, India; ^9^ Department of Biotechnology, Haldia Institute of Technology, West Bengal, India; ^10^ Department of Microbiology, All India Institute of Medical Sciences, Bibinagar, Hyderabad, India; ^11^ Genomix CARL Pvt. Ltd, Pulivendula, India; ^12^ Department of Computer Science, Flame University, Pune, India; ^13^ Cytogenetics Laboratory, Department of Zoology, Benaras Hindu University, Varanasi, India; ^14^ Department of Genetics, University of Alabama, Birmingham, AL, United States; ^15^ German Cancer Research Centre (DKFZ), Heidelberg, Germany; ^16^ Department of Applied Biology, Council of Scientific and Industrial Research-Indian Institute of Chemical Technology (CSIR-IICT), Hyderabad, India; ^17^ Amrita School of Biotechnology, Amrita Vishwa Vidyapeetham, Kerala, India

**Keywords:** SARS-CoV-2, COVID-19, ORFs, machine learning, co-morbidities

## Abstract

The year 2019 has seen an emergence of the novel coronavirus named severe acute respiratory syndrome coronavirus 2 (SARS-CoV-2) causing coronavirus disease of 2019 (COVID-19). Since the onset of the pandemic, biological and interdisciplinary research is being carried out across the world at a rapid pace to beat the pandemic. There is an increased need to comprehensively understand various aspects of the virus from detection to treatment options including drugs and vaccines for effective global management of the disease. In this review, we summarize the salient findings pertaining to SARS-CoV-2 biology, including symptoms, hosts, epidemiology, SARS-CoV-2 genome, and its emerging variants, viral diagnostics, host-pathogen interactions, alternative antiviral strategies and application of machine learning heuristics and artificial intelligence for effective management of COVID-19 and future pandemics.

## Origin, Taxonomy, and Hosts of Coronaviruses

Coronaviruses (CoVs) are single-stranded RNA viruses of size around 65-125 nm in diameter. Due to the presence of crown-like spike structure on the viral outer surface, they were named Coronaviruses (1). The CoVs are known to cause respiratory and gastrointestinal tract infections in humans, poultry, and animals (2, 3). These are a large group of viruses that belong to the order *Nidovirales*, family *Coronaviridae*, and subfamily *Orthocoronavirinae*, which in turn is divided into four genera: *Alphacoronavirus* (α-CoV), *Betacoronavirus* (β-CoV), *Gammacoronavirus* (γ-CoV) and *Deltacoronavirus* (δ-CoV) (4). Among seven humans CoVs identified to date including SARS-CoV-2, two of them belong to α-CoV (HCoV-NL63 and HCoV-229E) and the remaining five belong to β-CoV (HKU1, HCoV-OC43, SARS-CoV, MERS-CoV and SARS-CoV-2) (5, 6).

Coronaviruses are known for their ability to rapidly mutate, effectively cross the species barriers, and adapt to novel host environments (7). Out of the four genera, α-CoV and β-CoV are known to infect only mammals, while γ-CoV and δ-CoV primarily infect birds, with some reports indicating infections in mammals (8). Several studies have shown that bats, rodents, and avian species are natural reservoirs of diverse CoVs (9–12). For example, bats (*Rhinolophus* spp.) were identified as reservoirs of more than 30 CoVs including SARS-CoV (1, 13, 14). Currently, SARS-CoV-2 has been speculated to be transmitted to humans from bats through an unknown intermediate, which still needs to be conclusively proven (15). It is also possible that humans might have contracted an avirulent strain or a strain with lesser virulence directly or indirectly and then the virus might have undergone virulence-enhancing mutations resulting in human-to-human transmission (3). Whatever may be the causes for the origin of SARS-CoV-2, it remains to be proven with more supporting data.

Phylogenetic studies of SARS-CoV-2 revealed 79% nucleotide homology with SARS-CoV (16, 17) and 89% and 96% homologies with the two other SARS-like CoVs isolated from Chinese horseshoe bats *Rhinolophus sinicus* and *Rhinolophus affinis*, respectively (16, 18). However, these two SARS-like CoVs differ significantly in their receptor-binding domains. Upon comparison, SARS-like CoVs isolated from Malayan pangolins (*Manis javanica*) exhibited 85-92% nucleotide homology and stronger similarity in receptor binding domain with SARS-CoV-2 (19). Based on these findings, pangolins have also been considered as possible hosts in the emergence of SARS-CoV-2 (19). Besides pangolins, multiple species of wild or domestic animals like camels, mink, may also carry SARS-CoV-2 (8, 17, 20). Whether or not the suspected hosts are naturally infected by SARS-CoV-2 from humans remains to be proven.

## Diseased Phenotypes Associated With SARS-CoV-2

The SARS-CoV-2 infection causes multiple disease phenotypes such as pulmonary dysfunction, hematological alterations, inflammation, electrolyte imbalance, coagulation dysfunction, liver and kidney dysfunctions, cardiac muscle injuries (21). It has been surmised that due to coagulation dysfunctions, COVID-19 patients are at increased risk of venous and arterial thromboembolism (TE) and consequential mortality. These findings highlight the need to implement thromboprophylaxis protocols, while treating COVID-19 patients (22). However, more symptoms are still being discovered, some of which appear to be rare. Due to the diverse symptoms, it has been a challenging task to diagnose and manage the disease on time (23). Moreover, the impact of comorbidities (e.g., cardiomyopathies, hypertension, diabetes, etc.) on SARS-CoV-2 infection and disease progression makes the disease management even more challenging (24). Typically, symptomatic individuals with high body temperature pose a greater risk for transmitting the virus to others. However, such symptomatic individuals can be easily identified and isolated. On the other hand, the asymptomatic individuals may continue to remain unidentified through the regular screening procedures, thus jeopardizing the efforts to minimize viral transmission through identification and isolation of the virus-carrying individuals (25). Therefore, the existing precarious situation demands the identification and deployment of reliable serological markers to enable the identification of asymptomatic individuals (26). Application of *susceptible-exposed-infectious-removed* (SEIR) models to understand the COVID-19 epidemiology has estimated the R_0_ value of 2.03 for SARS-CoV-2, highlighting the importance of factors like hygiene, social distancing, and wearing PPE in reducing human-to-human and environment-to-human transmission of the virus (27). A recent meta-analysis by Heneghan et al. (28) indicated that firm conclusions cannot be drawn in the absence of recoverable viral culture samples of SARS-CoV-2. On the contrary, Greenhalgh et al. discuss reasons with supporting evidence to argue in the support of airborne transmission of SARS-CoV-2 (29). However, this is beyond the scope of this review as we limit the dissensions.

Over the past year, several studies have identified the presence of SARS-CoV-2 RNA in anal/rectal swabs and stool specimens of COVID-19 patients, even after the clearance of the virus in the upper respiratory tract (14, 30–34). With SARS-CoV-2 angiotensin-converting enzyme 2 receptor (ACE2) reported to be highly expressed in gastrointestinal epithelial cells (35, 36), it is suggested that the virus can actively infect and replicate in the gastrointestinal (GI) tract and thus has critical implications to the disease management, transmission, and infection control. In the past, symptoms such as diarrhea, nausea, vomiting and abdominal pain have been observed in patients infected with other coronaviruses such as SARS and MERS (37–39), indicating that the presence of the virus in the GI tract may be a common feature of the CoV infections. These studies collectively highlight the need for screening multiple clinical specimens from a single patient other than nasopharyngeal swabs, such as lung and tracheal aspirate, blood, pleural fluid, and fecal samples (40). Although different clinical specimens are of particular interest, whether or not this is done during the course of treatment of patients to ensure full recovery and no viral shedding is a subject of debate (41). Some evidence shows that SARS-CoV-2 can be vertically transmitted to the fetus or neonates from the infected mothers (42).

## SARS-CoV-2 Genome and Its Variants

**Figure 1:**
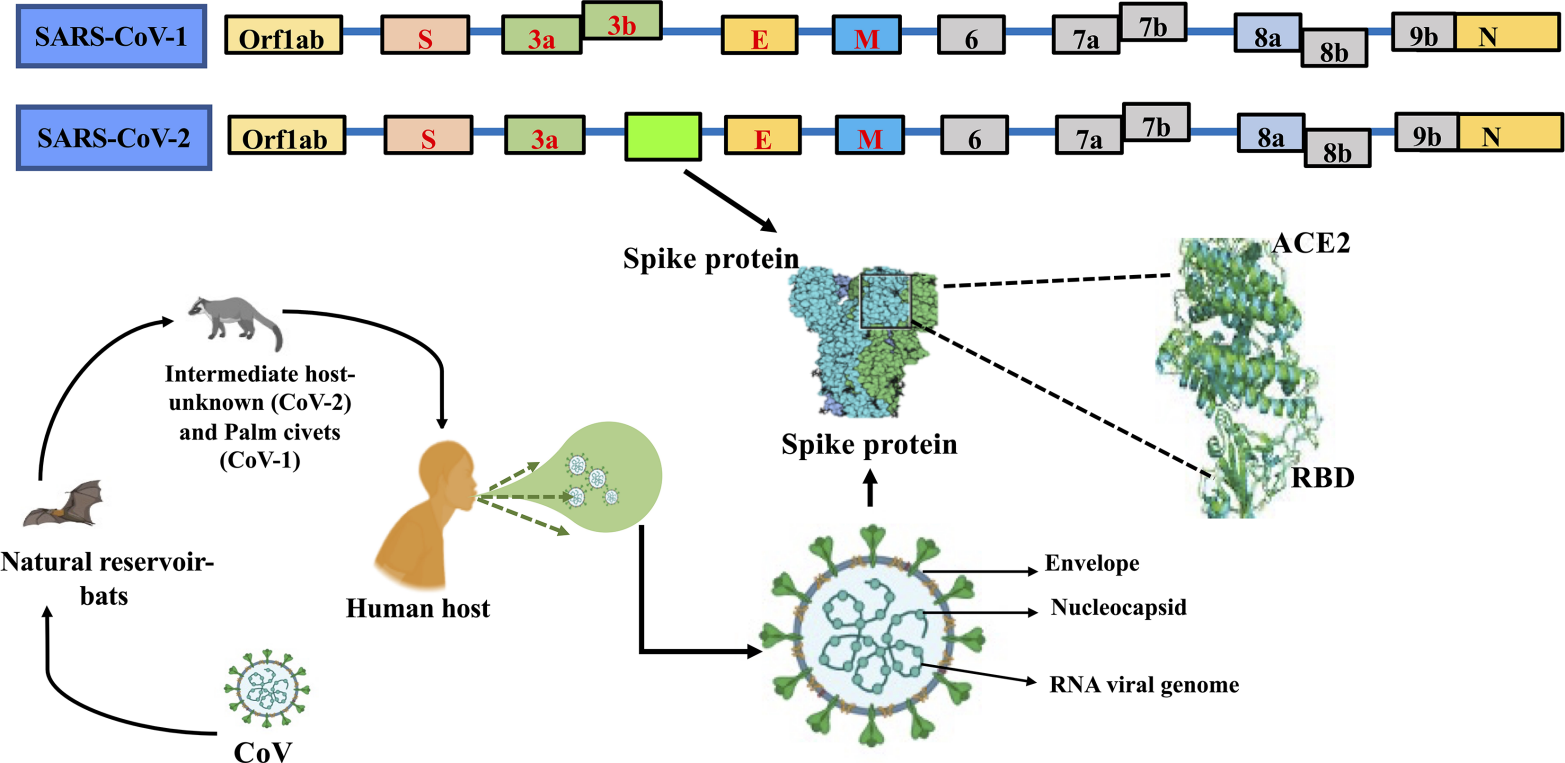
Genome comparison of SARS-CoV and SARS-CoV-2 along with ORFs depicting the common ancestry.

The SARS-CoV-2 carries the largest genome size of ~ 29.7 kb, which shares similarities with the genomes of other β-CoVs that have caused many epidemics in the past (43–50) (**Figure 1**). The SARS-CoV-2 consists of at least 16 non-structural and 4 structural proteins (51). The list of the genes encoded by SARS-CoV-2 genome and their known/predicted functions in the virus and the host is given in **Table 1**. After entering the host cell, the positive (sense) strand of viral RNA undergoes translation to synthesize non-structural proteins (nsps) from two protein-coding genes ORF1a and ORF1b (**Figure 1**). The CoV genome consists of six open reading frames (ORFs) of which the first ORF (ORF1a) encompasses about 2/3^rd^ of the genome and produces polypeptide 1a, which is further cleaved into 11 nsps. Due to ribosomal frameshift occurring upstream of ORF1a stop codon, translation of ORF1b yields another polypeptide called ORF1ab, which is further cleaved into 16 nsps. Cleavage of ORF1a and ORF1ab polypeptides is mediated by the virus-encoded proteases nsp3 and nsp5, which harbor papain-like domain and 3C-like domain, respectively. In addition, diverse forms of CoVs encode structural and accessory proteinS for example orf3 a/b protein, translated from the sub-genomic part of CoVs (52). Apart from the accessory proteins that have been found to play an important role in the viral-host interactions, several viral structural and accessory proteins, and a number of host proteins have been shown to interact with the viral RNA (74). Examples of host proteins that interact with the viral RNA include poly-A-binding protein, mitochondrial aconitase, pyrimidine-binding protein, and nuclear ribonucleoprotein which were known to be shown for SARS-CoV (75) and it is anticipated that that a similar interaction could be seen in SARS-CoV-2. However, this study is in infancy and beyond the scope of this review.

**Table 1:** Genes encoded by SARS-CoV-2 and their known or predicted functions.

Sl. No.	ORF/Protein Name	Type of protein	Gene size (in bp)	Role of the protein in viral replication and assembly	Role of the protein in host-poathogen interactions	The features that are unique to SARS-CoV-2 if any	Reference
1	ORF1a (codes for 16 nsps)	Non-structural	13218	Associated with gene expression/regulation, proteases involved in cleaving viral polyproteins, membrane rearrangement, RNA polymerase activities, proteins involved in viral replication (helicases, methyltransferases, exonucleases, etc.)	Involved in repression of host gene expression, disruption of host cell responses, dysregulation of autophagy.	NA	(52)
2	S (Spike protein)	Structural	3822	Helps in entry to host cells	Binds with ACE2 to gain entry into human cells. One of the most immunogenic viral antigens.	A furin cleavage site. A key protein being targeted for vaccine development. It carries several non-synonymous mutations in the RBD region and some in the hinge region. Collectively several mutations are predicted to enhnace binding of S protein with hACE2.	(53–55)
3	E (Envelope protein)	Structural	228	Participates in viral assembly, budding, envelope formation and pathogenesis	Interacts with several host cell proteins such as PALS1 disrupting tight junctions of epithelial cells and promotes viral spread. Also affects host cellular activities by interfering with endoplasmic reticulum (ER), Golgi and ER-Golgi intermediate compartment	Carries several non-synonymouos mutations which could play a role in enhanced viral pathogenesis; e.g., The changes resulted in Ser (56), Phe (57), Arg (58) and the C-terminal end (DLLV:72-75) are speculated to improve E protein interaction with tight junction-associated PALS1 which could play a critical role in SARS-CoV-2 pathogenesis and viral spread	(59–62)
4	M (Membrane protein)	Structural	669	Role in cell attachment and entry, viral particle assembly, and budding	Interacts with host cell proteins. Inhibits the production of type I interferon by blocking the formation of TRAF3.TANK,TBK1/IKKepsilon complex	Carries several non-synonymouos mutations which could play a role in viral pathogenesis.	(63)
	N (Nucleo-capsid)	Structural	1260	Protectis the genomic RNA of the virus, interacts with the M protein durin virion assembly and helps in viral transcription and assembly and budding.	Highly immunogenic, recognized as antigen in hosts. Associates with the host ER-Gologi complex. Modulates host cell cycle by regulating cyclin and cyclin-dependent kinase activities resulting in cell cycle arrest in S phase. Interacts with host translation elongation factor 1α (EF1α), suppressing host mRNA translation. Intereferes with interferon type I production and signaling. Antogonizes host anitviral RNAi responses.	Carries multiple non-synonymous mutataions. Some of these mutations might create a unique potential RNA binding pocket.	(64–66)
5	ORF6	Accessory	186	Role in viral pathogenesis.	Localizes at the nuclear pore complex and inhibits nuclear translocation of STAT1, antagonizing IFN-I signaling. Interacts with host cell proteins.	Not known yet	(67, 68)
6	ORF7a	Accessory	366	Not known yet, although it is under selective pressure with a possible role in host-switiching.	Interacts with host cell proteins and speculated to facilitate viral growth and replication. It is primarily localized to Gologi apparatus and also found on host cell surface. It interacts with Bcl-XL protein and induces apoptosis via caspase-dependent pathway. Inhibits antiviral mechanisms by inhibiting glycosylation of host protein BST-2 (also known as CD317)	Not known yet	(69, 70)
7	ORF7b	Accessory	132	Not known yet	Interacts with host cell proteins and speculated to facilitate viral growth and replication. Localized yo Gologi apparatus. Possseses leucine zipper motif with a potential to interfere with the functin of host cellular proteins which employ similar motifs.	Not known yet	(71)
8	ORF8	Accessory	366	Not known yet	It has ER-localization signal. Within the lumen of ER, it interacts with a vareity of host proteins and involved in inactivation of IFN-I signalling. It is presumably secreted out of host cells. ORF8 antibodies are one of the principal markers of SARS-CoV-2 infection.	SARS-CoV-2 has single ORF8 protein while SARS-CoV has ORF 8a and ORF 8b. It shares less than 20% similarity with SARS-CoV ORF8 sequences. Downregulates MHC-I in cells.	(72, 73)

Genome studies revealed that while SARS-CoV-2, SARS-CoV, and MERS-CoV share many similarities, they have multiple differences in their genomic and phenotypic structure which influence their pathogenesis (76). The RNA polymerases of most of the RNA viruses either lack or have a poor proof-reading activity (77). As a consequence, single-stranded RNA viruses mutate at a faster rate than DNA viruses, and as a result, genetic variants emerged as quasispecies (78). For instance, it was estimated that SARS-CoV can mutate its genome at the rate of 0.80-2.38 × 10^-3^ nucleotide substitutions per site per year which is similar to other RNA viruses (79). It is therefore anticipated that mutations at such rates allow the viruses to evolve faster, enabling them to escape host immune surveillance, develop resistance to drugs, vaccines and switch hosts. Moreover, the rapid rate of mutations could also increase the frequency of false negatives during nucleic acid-based viral diagnostics. Gustine and Jones have highlighted the role of a dysregulated innate immune response associated with hyperinflammatory syndrome in severe COVID-19 patients (80).

Several researchers have identified mutations in the SARS-CoV-2 genome with mutations identified in the SARS-CoV-2 genome that cause variants to become hotspots. Oster et al. have reviewed the trends in impending mutational hotspots ever since the covid infection rate has drastically been accounted for in various patients (81). Pachetti et al. identified eight novel recurrent mutations of SARS-CoV-2 in addition to 5 previously identified hotspots. Of the novel mutation hotspots, one was in the RdRp (RNA dependent RNA polymerase) gene involved in proof-reading machinery. This mutation was associated with a higher number of point mutations in Europe than viral genomes from Asia. The authors speculated that observed RdRp mutation could result in an enhanced viral replication, influencing mortality rates (56). analyzed 220 genome sequences from the GISAID database (www.gisaid.org) derived from patients infected by SARS-CoV-2 worldwide from December 2019 to mid-March 2020. The SARS-CoV-2 genome sequence available from the GenBank database was used as the reference in their study. They identified eight novel recurrent mutations of SARS-CoV-2, in addition to previously identified 5 hotspots. Of these 13 hotspots, 5 were predominantly present in the European isolates, and 3 were found exclusively in the North American isolates. Among the 13 hotspots, 6 were not observed in Asian isolates. It is assumed that the non-synonymous mutation (proline to leucine) in the RdRp gene could be affecting its proof-reading ability presumably by disrupting its interaction with the other protein cofactors such as the Exon domain of nsp14, nsp7, or nsp8, further alter the mutation rate of the virus (56). Further, they speculate that the observed RdRp mutation could result in an enhanced viral replication, influencing mortality rates. Several polymerase inhibitors are currently being tested in clinical studies targeting the RdRp protein of the virus. Some of these drugs have a predicted binding site in the SARS-CoV-2 RdRp hydrophobic cleft, which is adjacent to the identified mutation at the 14408 positions. Henceforth, it is possible that the observed mutation might also impart drug resistance to the virus, however, the proposed hypothesis needs to be experimentally proven.

As algebraic topology-based machine learning models were introduced to evaluate the SARS-CoV-2 spike glycoprotein (S protein) and host ACE2 receptor binding free changes, a number of mutations were identified (44). From the cluster analysis and the transmission dynamics, it is assumed that future SARS-CoV-2 mutations tend to have higher chances to mutate into significantly more infectious COVID-19 strains than the original one from Wuhan. Given the fact that the “infectivity-strengthening” mutations spread faster than “infectivity-weakening” mutations, the study concluded that the proportion of asymptomatic cases have drastically increased despite impending cases in South Korea (82). In another study, phylogenetic analysis of ~1,400 SARS-CoV-2 genomes isolated from India yielded 7 clusters, of which one was unique to India. This unique cluster (Clade I/A3i) included three variants; C6312A, C13730T, and C28311T, which resulted in amino acid changes T2016K (orf1a), A97V (RdRp) or A88V (orf1b) and P13L (N protein), respectively (83). These changes are hypothesized to enhance the virulence and/or infectivity of the virus. The mutation A97V in the RdRp protein is located in its NiRAN domain, suggested to be relevant in RNA binding and nucleotidylation activity (84). Mutations have also been identified in other viral proteins with potential functional consequences (57, 85, 86). However, the impact of all these mutations on the functions of the respective proteins and their consequences on viral pathogenesis needs to be experimentally tested. Among the recently identified variants of SARS-CoV-2, the one carrying D614G mutation in the spike protein of the virus has emerged as the most prevalent variant in the global pandemic, possibly due to its greater fitness advantage (87). This variant was found to be more infectious resulting in a higher viral titer in patients. In addition to viral variants, a recent genome-wide association study (GWAS) in the UK identified eight human genetic variants consisting of a combination of alleles at multiple loci that are predicted to increase the risk of mortality among COVID-19 patients (88). Lately, some of the SARS-CoV-2 variants with mutations in the S protein are behind the surge in the second wave of SARS-CoV-2 infections in many countries including India. Among the three sub lineages of B.1.617, namely B.1.617.1, B.1.617.2, and B.1.617.3, the variant named B.1.617.1.2 has been found to rapidly spread in many countries including India. Recently, B.1.617.1.2 was classified by WHO as a ‘variant of concern’ based on evidence showing higher transmission rates and reduced neutralization by antibodies obtained from the convalescent serum of infected or vaccinated individuals (89). Similarly, many other variants are being identified globally and being monitored for their epidemiology.

## Variants of Interest/Concern

Several variants have been identified globally, which appear to spread more quickly, potentially increasing COVID-19 infections. Based on the factors, including the severity of the virus and their ability to spread, four variants viz. B.1.1.7, B.1.351, P.1 and B.1.617.2, also known as alpha, beta, gamma and delta, respectively, have been characterized as the variants of concern (**Table 2**). An alpha variant, identified initially in the United Kingdom and delta variant, first identified in India, is outspread with a much faster rate than beta and gamma variants, first determined in South Africa and Japan/Brazil, respectively. Hence, alpha and delta variants may potentially cause more sickness and increase the number of deaths globally. On the other hand, treatment with monoclonal antibodies is less effective against beta, gamma, and delta variants, while treatment for alpha variants is known to be effective (101). These variants have different alterations in the spike protein leading to increased susceptibility, virulence and transmission that may affect viral replication and host immune response. More recently, a novel South African variant C.1.2 was identified which would escape antibody response. BNT162b2 (Pfizer/BioNTech) and mRNA-1273 (Moderna) use a formulated vaccine which is used to elicit potential response against the aforementioned SARS-CoV-2 variants thereby combating rapid diagnosis (102).

**Table 2:** List of known COVID-19 variants of concern (6, 55, 59–74, 90–100).

WHO label	Lineage	First identified	Rate of outspread	Monoclonal antibody treatment
Alpha	B.1.1.7	United Kingdom	Much faster; increased number of deaths	Effective
Beta	B.1.351	South Africa	May spread faster	Less effective
	B.1.351.2			
	B.1.351.3			
Gamma	P.1	Japan/Brazil	Faster	Less effective
Delta	B.1.617.2	India	Much faster; increased number of deaths	Less effective
	B.1.617 or B.1.617.1	India		
	AY.1			
	AY.2			
	AY.3			
Epsilon	B.1.427/429	California		
Eta	B.1.525			
Lota	B.1.526			
New beta variant	C.1.2	South Africa Now spreading across Europe and New Zealand	Rapid and fastly increasing.	

## Host-Pathogen Interactions, Co-Morbidities and Immune Response

The infection starts with the binding of virus elements to the host cell surface receptors, followed by viral entry and multiplication. Moreover, the binding of virus to the host receptor is primarily required for viral transmission to other host species as well (103). Most of the human CoVs (HCoVs) have been ascertained to recognize protein peptidases as their receptors except HCoV-OC43 and HKU1 which utilize sugar molecules for cellular attachment (104). For example, the HCoV-229E binds to human aminopeptidase N (105), while MERS-CoV binds to human dipeptidyl peptidase 4 (58). On the other hand, SARS-CoV and HCoV-NL63 interact with ACE2 for viral entry into the host (106). In CoVs, the passage of viral entry into the host is mediated by the spike (S) glycoprotein, situated at the viral surface 71. The S protein is cleaved by human proteases into S1 and S2 subunits, which are involved in receptor identification and cell membrane arrangement, respectively (53). The N-terminal and the C-terminal domains (NTD and CTD) of the S1 subunit play an important role in the functioning of receptor-binding domains (RBD) in SARS-CoVs and MERS-CoVs (58, 107). The crystal structures of HCoV-NL63 and SARS-CoV RBD interactions with human ACE2 (hACE2) receptors are already known (107, 108). The composite crystal structure of interaction between S1 CTD of SARS-CoV-2 and hACE2 receptor has revealed that most of the binding residues of SARS-CoV-2 in hACE2 show similarity with SARS-CoV binding sites (90). Furthermore, the crystal structures of trimeric S protein of SARS-CoV-2 published recently with buried and exposed RBD regions have also been found to be consistent with structural characteristics of S proteins in MERS-CoV and SARS-CoV (91). While receptor recognition allows us to identify hCOV pathogenic determinants, the ACE-2 Receptor recognition is an important determinant of hCoVs infection and pathogenesis. Comparable to SARS-CoV RBD, it was assumed that SARS-CoV-2 RBD is less potent and exposed given the spike proteins’ switching between lying down and standing up positions (109). The former buried positions were in turn larger and so the masking regions are favored by immune evasion of spike proteins’ through ACE-2. In summary, SARS-CoV-2 has diffident buried and exposed surfaces affecting the chemistry of pathogenic determinants besides host protease activation.

**Figure 2:**
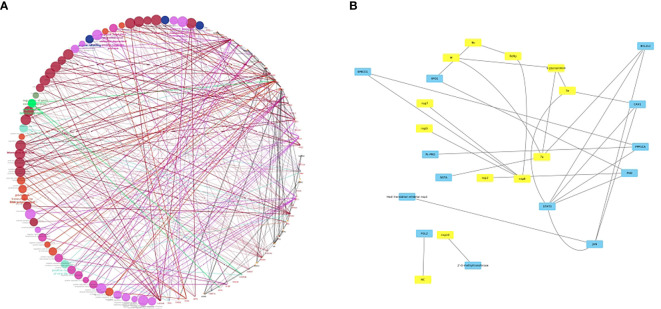
**(A)** *bona fide* network analysis of HCoV-host interactome network. A subnetwork illustrating the HCoV-host interactome where colored nodes represent diverse enriched pathways in host proteins and edge colors represent evidence collected from protein-protein interactions. The Figure was generated using Cytoscape (111) with the host proteins shown to be interacting with the viral conglomerate proteins. The pink edges indicate the experimentally validated interactions, with green, grey, orange, and maroon edges representing fusion, subcellular location, coexpression and associations from text mining. **(B)** A sub-figure with all the viral proteins in yellow colored nodes and the host proteins in blue colored nodes. This figure is depicted keeping in view of the interplay of various host-pathogen interactions. A **Supplementary Table 1** is added which shows the list of interactants based on the text mining, co-expression network and overall scores. However, as no co-expression network studies have been deciphered yet between virus and the host, the network is distributed largely based on the text mining interactions. In conclusion, the network largely explores how the SARS-CoV-2 proteins have an interplay with a lot of host proteins, viz. SPECC1, a mitochondrial like protein, PHB, a prohibitin associated with cancer and FGL2/fibroleukin prothrombinase, a protein associated with clotting factors and alveolar macrophage activation.

Furthermore, the newly evolved delta mutations in SARS-CoV-2 may impact disease severity and become immunocompromised. As it is known that the variant replication is manifold when compared to its predecessor variants, whether or not vaccinated people have the highest chance to skip the infection and transmission is under evaluation (110). This could be due to neutralizing antibodies that might have evaded the infection. These delta variants host multiple mutations in the S1 subunit, including the RBD that seem to have concurrent epitope tags. Therefore, the knowledge of the crystal structure of interactions between host and viral proteins will not only allow repurposing of the existing drugs but also the discovery of new antiviral agents. As a prelude to this, we have developed a *bona fide* network of HCoV-host interactome networks which could be used to identify key putative candidate interacting pairs responsible for the viral pathogenesis (**Figure 2**). Among HCoVs, different viral proteins are being used for viral entry. For example, β-CoVs use their S protein for cell binding and entry. SARS‐CoV binds to ACE2, MERS‐CoV utilizes dipeptidyl peptidase 4 (DPP4) of the host. Recent modelling of the structure of SARS‐CoV‐2 S protein predicts that it can bind to both human DPP4 and hACE2 (112).

The diabetic patients appear to be highly vulnerable to SARS-CoV-2 infection. Given the administration of angiotensin-directed medications for diabetics, a new treatment regimen is being suggested for COVID-19 patients with diabetes (113, 114). Lately, diabetic patients have shown an alarming increase in a post-covid complication called mucormycosis, which is a highly invasive opportunistic fungal infection affecting several vital organs of the body including, the brain, eyes, ear, nose, throat and mouth (115). Mucormycosis is caused by a group of fungi called Mucormycetes (also popularly known as black fungus) and the infection has become one of the factors involved in increasing COVID-19-associated morbidity and mortality (116). The use of steroids to reduce inflammation in COVID-19 patients leads to a further increase in blood sugar levels and reduction in immunity, further increasing the susceptibility to opportunistic black fungus infections (115). In addition to diabetes, other factors which further enhance sensitivity to infection are smoking, age, and obesity (117). Similarly, hospital-acquired nosocomial infections pose additional challenges in treating COVID-19 patients, underscoring the need for early diagnosis of these opportunistic pathogens. Pulmonary dysfunction, asthma, and bronchitis due to air pollution have also been reported to increase the chances of SARS-CoV-2-associated morbidity and mortality (118). In this context, mathematical modeling and artificial intelligence can serve as valuable tools in predicting the impact of various environmental variables and comorbid conditions on the progression of SARS-CoV-2 infection and *vice versa* (119). Given the multifaceted involvement of ACE-2 in SARS-CoV-2 biology and its role in comorbid conditions, there is a need to understand the association of ACE-2 expression with patient fatality rate.

Over the last year, multiple studies reported an association between the ABO blood group and COVID-19 (120–124). All these studies concur that individuals with blood group A exhibit a higher risk of SARS-CoV-2 infection, morbidity and mortality. On the other hand, individuals with blood group O exhibit a lower risk for the same parameters. *In vitro* studies by Guillon et al. (54) demonstrated inhibition of adhesion of SARS-CoV-expressing cells to ACE2-expressing cells by anti-A antibodies, which might explain why individuals with blood group A showed higher susceptibility to COVID-19 infection. However, these findings need to be further validated before blood groups can be used as prognostic markers.

Lower levels of certain vitamins such as vitamin D, A, and K also appear to influence SARS-CoV-2 infection and disease progression. Given the multifarious roles of these vitamins in the maintenance of mucosal membrane barriers, innate and adaptive immunity, maintenance of blood pressure and blood glucose levels, integrity and functioning of skeletal and non-skeletal tissues among other functions, their deficiency is expected to be a potential risk for SARS-CoV-2 infection and disease severity (125–131).

## Does SARS-CoV-2 Hijack Mitochondria to Induce Infections?

Mitochondria have been known to be involved in inducing innate immune responses primarily against viral attacks (132). Upon viral infection, damage to mitochondrial membrane results in leakage of the mitochondrial DNA into the cytosol. In addition to the viral genome, the presence of mitochondrial genome in the cytosol is sensed by cytosolic surveillance systems that recognize these cytosolic DNA called Danger Associated Molecular Patterns (DAMPS) (133). Recognition of DNA by cytosolic DNA receptors triggers immune response and inflammation leading to the recruitment of macrophages and dendritic cells. Hence, any alteration with the mitochondrial membrane during infection may lead to accelerated immune response and explain the observed clinical symptoms during COVID-19. The ACE2 receptor and TMPRSS2, a transmembrane serine protease, both used by hCoVs to enter host cells, can alter mitochondrial function, which might enable the virus to hijack mitochondria to its advantage in facilitating its spread to the neighboring cells. We have earlier shown that mutations in the SARS-CoV-2 may manipulate mitochondria even as ACE2 is regulated by its inherent mitochondrial function (132). Moreover, the 5’ and 3’ UTR regions of the viral transcript have been found to have mitochondria localization signals (51). Several ORFs like ORF9b, ORF3b, ORF7a, and ORF8b of the SARS-CoV are also shown to be localized in the host mitochondria. These sequences have high similarity with those found in SARS-CoV-2, indicating that it is highly likely that SARS-CoV-2 transcripts may possess the ability to localize in host mitochondria too. Several SARS-CoV proteins have been known to interact with host mitochondrial proteins and yet, the knowledge remains limited regarding the cellular significance of these interactions. CoVs have been known to reside in ER-derived double membrane vesicles to avert host immune responses and therefore CoVs might have been adopted to reside in mitochondria-derived vesicles for similar purposes. These observations suggest that mitochondrial hijacking may be an essential mechanism in SARS-CoV-2 infection and therefore, drugs that selectively restore mitochondrial function and promote its biogenesis may prove to be effective anti-inflammatory agents in the treatment of COVID-19. Infection of host cells by SARS-CoV-2 induces a strong immune response which could be linked to the ability of the virus to use the host organelles such as endoplasmic reticulum (ER) for its replication. These observations further emphasize the need to understand the crosstalk between the viral proteins and the proteins of mitochondria and ER and its influence on the formation of mitochondria-derived double-vesicles (MDV) and mitochondrial antiviral-signaling proteins (MAVS), which induce apoptosis. Studies are underway to know whether or not the non-structural proteins (nsps) are an integral part of this mechanism (51). While it is also not clear how the RNA from the virus enters the mitochondria in human cells, it is hypothesized that it may interact with ACE-2 receptors in regulating mitochondrial function. Higher levels of ACE-2 have been shown to restore impaired mitochondrial function (134). As SARS-CoV-2 infection leads to mitochondrial hijacking, it negatively impacts cellular bioenergetics leading to asphyxiation, further causing fatalities. It will be interesting to know how the mutations in mitochondrial genomes in humans might contribute to diverse responses to SARS-CoV-2 infections, akin to the GWAS study conducted in the UK which identified a set of alleles associated with increased risk of mortality among COVID-19 patients (88). An epilog to this, we have also recently hypothesized how SARS-CoV-2 transgressing non-coding RNAs esp lncRNAs of the host may allow us to understand the known unknown regions of the viral genome (110).

## Disease Management of COVID-19: Potential Treatment Options and Repurposing the Existing Drugs for COVID-19 Treatment

Since the onset of COVID-19, several attempts have been made to identify or develop new antiviral agents or to repurpose existing drugs to antagonize the virus. Among the antiparasitic agents, hydroxychloroquine (HCQ), chloroquine, and ivermectin were extensively tested to repurpose them against SARS-CoV-2. While some studies showed promising results, others were inconclusive (135). Several antiviral compounds that are known to act against viruses including CoVs have been explored to repurpose them against SARS-CoV-2. Among them, some of them with the most promising outcomes include, Remdesivir (GS-5734), Favipiravir, Lopinavir, and Ritonavir. Remdesivir (GS-5734), an analog of adenosine, is a broad-spectrum antiviral agent that gets incorporated into nascent RNA, resulting in premature termination of RNA synthesis (136–138). Favipiravir is another FDA-approved drug for the influenza virus that showed antiviral activity against SARS-CoV-2 *via* the inhibition of viral RNA polymerase (139). The HIV protease inhibitors Lopinavir and Ritonavir have been under clinical focus for their known roles in inhibiting 3C-like proteases [3CL^pro;^a cysteine protease that hydrolyses the Viral proteins (hCoVs)] of CoVs with which SARS-CoV-2 shares up to 96% sequence identity (140). However, recent clinical trials failed to show any significant benefits of these anti-HIV drugs against SARS-Co V-2 (141).

Additionally, some of the herbal medicines are being used or evaluated to treat COVID-19 based on findings from clinical trials or *in vitro* studies. For example, the traditional Chinese medicine made up of Qingfei Paidu Decoction (QFPD) accelerated the recovery from COVID-19 symptoms and reduced the mortality rates (142–144). Earlier, QFPD had also been shown to be effective against SARS-CoV (145). Therefore, QFPD is currently being used as one of the adjunct treatments against COVID-19 in China. Similarly, tryptanthrin, a compound isolated from the leaf of the Chinese herb, *Strobilanthes cusia*, has been explored as another treatment option since it displayed an antiviral activity against human coronavirus NL63 in a cell-type independent manner *in vitro* (146). In addition, several active phytoconstituents obtained from medicinal plant species such as *Curcuma longa* (turmeric), *Withania somnifera* (Ashwagandha), *Tinospora cordifolia* (Giloy), and *Ocimum sanctum* (Tulsi) have been subjected to molecular docking studies to identify compounds with a potential to interact with and inhibit SARS-CoV-2 proteins (147–149). Some of these compounds have shown promising results and are being pursued further.

Various computational tools have been employed to rapidly identify potential new drugs and the existing drugs for treatment of COVID-19 (150). Artificial intelligence (AI) has been deployed for predicting the structure of SARS-CoV-2 proteins which will help in identifying new drugs besides repurposing the existing drugs to treat the virus. For example, Beck et al. (151) developed a deep learning-based pretrained drug-interaction model called *molecule transformer-drug target interaction* (MT-DTI) to shortlist commercially available drugs for their potential to target SARS-CoV-2 viral proteins. Their findings predicted that Atazanavir, an anti- HIV drug to possess the highest inhibitory potency. Their study also predicted other drugs including Remdesivir, Efavirenz, Ritonavir, and Dolutegravir to have anti-SARS-CoV-2.

Molecular docking studies have been extensively used to identify molecules that can bind to the SARS-CoV-2 machinery and inhibit its replication (152). Docking studies of nigelledine and hederin from *Nigella sativa* with SARS-CoV-2 main protease called 3CL^pro^ (M^pro^), showed improved docking score for ligand binding free energies compared to Chloroquine, HCQ and Favipiravir. Therefore, these drugs have been provisionally considered as potential therapeutic agents for treating SARS-CoV-2 (153). In another study, a chemographic analysis of anti-CoV structure-activity information from a public database (ChEMBL) containing a vast pool of 800 million organic compounds, about 380 potential anti-CoV agents were identified, which needs to be experimentally tested and validated for their antiviral activity against SARS-CoV-2 (154).

Elfiky (155) built a model for the viral RdRp to test its binding affinity to some of the clinically approved drugs and drug candidates by carrying out molecular modeling, docking, and molecular dynamics simulations for the viral protein RNA-dependent RNA polymerase (RdRp). In addition to Sofosbuvir, Ribavirin, Galidesivir, Remdesivir, Favipiravir, Cefuroxime, Tenofovir, and Hydroxychloroquine showing effectiveness, new derivatives were shown to have promising results for the attachment to the SARS-CoV-2 RdRp. Recently, the crystal structure of SARS-CoV-2 3CL^pro^ (Liu et al., 10.2210/pdb6LU7/pdb) was used to screen several approved drugs or drugs in clinical trials *via* virtual docking (156). The findings from this study predicted several promising drugs to inhibit the 3CL^pro^. The list includes Carfilzomib (a proteasome inhibitor being used as an anticancer drug), Eravacycline (a florocycline- a synthetic analog of tetracycline), Valrubicin (an analog of doxorubicin which is an inhibitor of nucleic acid metabolism, being used to treat bladder cancer), Lopinavir (HIV-1 protease inhibitor), Elbasvir (anti Hepatitis C viral drug, which targets nsp 5A), and Streptomycin (a known antibiotic that inhibits bacterial protein synthesis). In a similar docking study, along with HIV protease inhibitors (Lopinavir, Asunaprevir, Indivavir, and Ritonavir), new molecules including Methisazone (an antiviral drug that inhibits mRNA and protein synthesis in poxviruses), CGP42112A (an angiotensin AT2 receptor agonist), and ABT450 (Paritaprevir: an antiviral drug that inhibits nsp 3-4A serine protease of HCV) were predicted to bind and inhibit 3CL^pro^ of SARS-CoV-2 (157).

Furthermore, synergistic studies carried out on Lopinavir, Oseltamivir, and Ritonavir showed that when used together these drugs exhibited greater binding affinity and inhibition of the viral protease proteins than when used alone (158). In another docking study, ~ 1.3 billion compounds from the ZINC15 database were docked against the active site of the SARS-CoV-2 3CL^pro^ using the Deep Docking (DD) platform, which provides a fast prediction of docking scores (159). The authors identified the top 1000 potential ligands for SARS CoV-2 3CL^pro^. In a similar study, the potential drugs from the ZINC15 database were screened for their binding affinity for S protein and 3CL^proo^ of SARS-CoV-2. The findings from this study identified Zanamivir (an anti-influenza drug), Indinavir and Saquinavir (anti-HIV drugs), Remdesivir (anti-SARS-CoV and an anti-ebola virus drug) as compounds with high binding affinities for 3CL^pro^. In addition, flavin adenine dinucleotide (FAD) adeflavin, coenzyme A, tiludronate, and Dpnh (NADH) were predicted to bind the S protein with high affinity (160). Recently, 2 deoxy glucose (2DG), an anticancer drug, has been approved for emergency use in India as an adjunct therapy to treat COVID-19. 2DG inhibits host glycolytic pathway, anti-inflammatory action and interacts with viral proteins (161, 162). An exhaustive list of repurposed drugs targeting various stages of virus life cycle and their current status of clinical trials has been listed in Almasi and Mohammadipanah (163).

## Machine Learning Heuristics and Artificial Intelligence for COVID-19 Management

The artificial intelligence (AI) and machine learning (ML) paradigms have offered effective tools and algorithms to combat COVID-19 pandemic. AI is being successfully applied for disease cluster identification, monitoring of COVID-19 patients, in determining mortality risk, disease diagnosis and management, contact tracing through geotagging, resource allocation, facilitating training of health care personnel, data management, and in predicting future pandemic outbreaks and the disease trend (164–166). The power of AI-ML in viral diagnostics and the management of COVID-19 have been summarized in various reviews (167–170) (**Table 3**). Clinically, computed tomography (CT), positron emission tomography-CT (PET/CT), lung ultrasound, and magnetic resonance imaging (MRI) are being used in COVID-19 diagnosis. The AI can supplement medical imaging-based COVID-19 diagnosis particularly reducing the diagnosis time (167, 206). Application of deep learning to X-ray and CT scan imaging has resulted in detection of COVID-19 with high accuracy, sensitivity, and specificity (207, 208). Such applications also effectively differentiated symptoms due to COVID-19 from bacterial pneumonia. In another novel study, AI was applied to predict the outcome of the RT-PCR-based diagnosis of COVID-19 on the basis of 16 simple parameters derived from complete blood profile (205).

**Table 3:** Summary of diagnostic methods used to detect SARS-CoV-2 (171–204).

	Name of the method	Salient features/advantages	Disadvantages	Time taken for detection from the time of sample collection	Feasible for pooled testing?	References
**1. Detection of RNA**	RT-PCR	Gold standard method for screening and diagnosis in the early phase of infection. Advantages include automation, high-throughput analysis and relatively reliability. Also determines relatively viral load.	Higher occurrence of false positives and false negatives, requires thermocyclers, laboratory set up and expertise to carryout the test and the analysis. Lack of symptoms in positive individuals; Highly vulnerable for errors due to cross contamination during sample collection, processing and while performing the test. High chance of laboratory error during sampling Need of skilled personnel	~ 4-6 hours	Yes	(32, 176, 188, 205)
	RT-LAMP	Performed using isothermal amplification. Advantages: It does not require specialized laboratory equipment (e.g. thermocyclers). Can be performed at a wide range of pH and temperature. Does not require thermocyclers, faster test results, easy to use, cost-effective, sensitivity comparable to RT-PCR Colorimetric visualization of results; Non-processed samples can be assayed.	Requires more number of primers and thereby increases chances of primer dimer formation contributing to false positives. For the same reason, primer designing is challenging. Multiplexing could be problemative. Less versatile than PCR .	<1 h	Yes	(172)
	RT-RPA	Performed using isothermal amplification. Advantages: It does not require specialized laboratory equipment (e.g. thermocyclers). Can be performed at a wider range of temperatures. Does not require thermocyclers, faster test results, easy to use, cost-effective, sensitivity comparable to RT-PCR Colorimetric visualization of results; Non-processed samples can be assayed.	Primer and probe design for RPA are less established; Less sensitive than RT-PCR and RT-LAMP. Requires higher concentrations of dNTPs.	<1h	Yes	(198)
	RNA NASBA	Employs reverse transcription followed by amplification of RNA by T7 RNA polymerase followed by detection of the amplified RNA using fluorescent molecular beacon DNA probe. It is an isothermal reaction (41°C) and has faster amplification kinetics compared to RT-PCR and RT-LAMP; Does not require thermocyclers. Compatible for multiplexing and high throughput analsysis.	Low temperature of the reaction conditions increasese the chances of non-specific primer interactions. Except one, NASBA enzymes are heat-labile, requiring the addition of these enzymes after the melting step. Since the primers used are not incorporated in the amplicon and therefore labeled primers can't be used for detection.	90 minutes	Yes	(179, 185)
	SHERLOCK	Viral RNA is reverse transcribed first to produce cDNA following which fluorescently-labeled single-stranded DNA (ssDNA) reported probes are indiscriminately cleaved either through FnCas9- or Cas12a-sgRNA complex. Alternatively synthesized cDNA can be used for *in vitro* transcription and the RNA thus produced will acticate nuclease activity of Cas13a, resulting similar cleavage of fluorescently-labeled ssDNA reported probesor Cas12based. All components of SHERLOCK can be freeze-dried; Highly sensitive and specific. Capable of detecting single target RNA/DNA molecule. Amenable for multiplexing and is one of the rapid nucleic acid detection method.	Multi-step detection, protocol optimization is challenging. Cas13a depends on intact *in vitro*-synthesized RNA and therefore proned to challenges associated with RNA degradation.	~ 40-90 minutes	?	(186)
**2. Detection of viral antigen**	Rapid antigen test	Antibodies specific to vrial antigens are used to detect the virus. One of the most rapid methods, easy-to-perform and interpret, requiring no specialized equipment or expertise. Most suitable for point-of-care diagnostics.	It takes weeks or months to produce high-titer antibodies and usually they are not as sensitive and specific as nucleic acid-based approaches. Often, confirmation of the negative results by rapid antigen test requires RT-PCR to rule out infection.	~15 minutes	No	(195)
**3. Detection of antibodies generated in response to SARS-CoV-2 infection**	Lateral flow assays	Fast detection of IgM/IgG antibodies specific for SARS-CoV-2 proteins. Direct detection from plasma, serum, whole blood or fingertip blood and requires less amount of sample. Instruments, specialized expertise not required. Amenable for self-testing at home as well as for point-of-care testing centres. Long shelf -life.	Often, confirmation of the negative results by rapid antigen test requires RT-PCR to rule out infection. Requires highly purified antigens for accuracy.	~15 minutes	No	(175, 187, 190, 193)
	ELISA (IgA, IgM and IgG)	One of the most commonly used methods to detect both antigen/antibodies. Hihgly sensitivity in detecting antiviral antibodies. Allows highthroughput analysis.	Clinical performance data are scarce; Cross-reactivity has been reported; Cost per test is high as compared to other tests	2-3 h	Yes	(181)
**4. Detection of Symptoms**	Chest computed tomography (CT) Scan	Chest CT scans help if identifying characteristic lung pathology associated with the disease such as ground glass opacity, bilateral consolidation, interlobular septal thickening and pleural effusion. Low false-negative rate. Can be used to monitor the progression of the disease/recoverey.	Requires expensive equipment, proper lab facility and techincal expertise to carry out the test and analyze the results. It requires physical presence of the suspected individuals/patients. Diagnosis is not specific to the pathogen. Imaging protocols vary across locations	~1h	No	(206, 207)
	Radiography (X-ray)	Radiography (X-ray) identifies unilateral and bilateral infilterate. Rapid, low false-negative rate. Can be used to monitor the progression of the disease/recoverey. The cost is lower than CT but at the same less-sensitive and is more useful in monitoring the disease during later stages.	It is less sensistive and demostrates limited pathological features associated with the disease. Generally it cannot detect early stages of the disease. Requires expensive equipment, proper lab facility and techincal expertise to carry out the test and analyze the results. It requires physical presence of the suspected individuals/patients. Diagnosis is not specific to the pathogen.	~ 45 minutes	No	(208)

Randhawa et al. (209) identified an intrinsic SARS-CoV-2 virus genomic signature in combination with a machine learning-based alignment-free approach for a fast, scalable, and highly accurate classification of whole SARS-CoV-2 genomes. They coupled supervised machine learning with digital signal processing (MLDSP) for genome analyses. Moreover, the authors claimed highly accurate real-time taxonomic classification of SARS-CoV-2 genomes simply based on raw DNA sequence, without any requirement of specialized biological knowledge or training or gene/genome annotations. Their method is extremely rapid; for example, analysis of a data set consisting of 5538 unique viral genomes with a total of ~ 62 Mb was carried out within a few minutes with high classification accuracy. Other applications of ML and AI in protein structure prediction, identification of potential drugs using molecular docking studies, reverse vaccinology, automating the data collection and transmission using cyber physical systems (CPS) from the current and futuristic diagnostic devices (e.g., lab-on-chip) are described in previous sections.

## Conclusions

COVID-19 undoubtedly has turned out to be one of the most destructive pandemics in recent history having a negative impact on all aspects of human life. The repetitive global resurgence of apparently more infectious strains of SARS-CoV-2 has made the pandemic unrelenting. We have attempted to summarize the various critical findings pertaining to COVID-19 biology from detection to treatment. Given the fatic paradigm, there is a need for the scientific community to apply Thoughts-Action-Debate (TAD) applying *Trikarna shuddhi*, a Sanskrit adage of learning as we say “ome” to prepare for future pandemics.

## Author Contributions

PSu and PBK conceptualized the idea for the review. The authors with + sign contributed equally, performed the literature search, analyzed cited references, and wrote the original article. PSu, ORB, PBK, AKS, HKP, KKS, GC critically revised the article. All authors contributed to the article and approved the submitted version.

## Conflict of Interest

RP was employed by Genomix CARL Pvt. Ltd.

The remaining authors declare that the research was conducted in the absence of any commercial or financial relationships that could be construed as a potential conflict of interest.

## Publisher’s Note

All claims expressed in this article are solely those of the authors and do not necessarily represent those of their affiliated organizations, or those of the publisher, the editors and the reviewers. Any product that may be evaluated in this article, or claim that may be made by its manufacturer, is not guaranteed or endorsed by the publisher.
